# Preparation of Mechanically Anisotropic Polysaccharide Composite Films Using Roll-Press Techniques

**DOI:** 10.1021/acsomega.2c07077

**Published:** 2023-02-01

**Authors:** Takuya Sagawa, Yuichi Nikaido, Kazutoshi Iijima, Masahiro Sakaguchi, Yusuke Yataka, Mineo Hashizume

**Affiliations:** † Department of Industrial Chemistry, Faculty of Engineering, Tokyo University of Science, 6-3-1 Niijuku, Katsushika-ku, Tokyo 125-8585, Japan; ‡ Graduate School of Chemical Sciences and Technology, Tokyo University of Science, 12-1 Ichigayafunagawara-machi, Shinjuku-ku, Tokyo 162-0826, Japan; § Graduate School of Engineering, Tokyo University of Science, 6-3-1 Niijuku, Katsushika-ku, Tokyo 125-8585, Japan

## Abstract

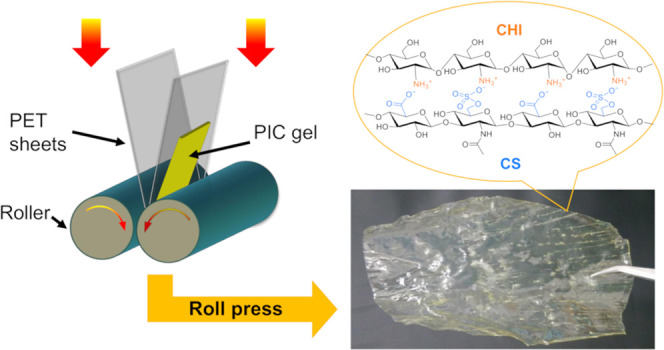

Natural polysaccharides are biocompatible and biodegradable; therefore, they can be used as feedstock for biodegradable structural materials and biomaterials. In this study, anisotropic polysaccharide composite films consisting of chondroitin sulfate C (CS) and chitosan (CHI) were fabricated from their polyion complex (PIC) gels by roll-press techniques. The obtained films (CS/CHI films) were thin and transparent, similar to the composite films prepared by hot-press techniques. The roll-press conditions were optimized, and it was observed that the molecular weight of CHI did not significantly affect the formability of the films, whereas the roll temperature and rolling speed were important. The tensile tests of the roll-pressed films revealed that the mechanical strength of the films in the mechanical direction (MD) was approximately 5 times higher than that in the transverse direction (TD), indicating that the roll-press techniques imparted mechanical anisotropy to the films. Additionally, the films shrank in the MD and expanded in the TD after immersion in aqueous solutions, followed by drying. Such anisotropic shrinking and expanding properties indicate that these films can be used as shape-memory materials.

## 1 Introduction

Materials made by assembling macromolecules have an advantage: a series of materials with different physical properties, such as mechanical strength, can be obtained without the chemical modification of the polymers, depending on the features of the assembly (assembling states).^1,2^ If natural polymers can be used for such purposes, natural resources can be effectively used, thereby expanding their versatility as biocompatible materials.^3−7^ In this regard, natural polysaccharides are promising polymers because of their abundance in nature. For example, glycosaminoglycans, a series of acidic polysaccharides, are promising biomaterials because of their physical and biochemical properties.^8−15^

When fabricating structural materials for industrial and biomedical applications, the inherent water solubility of polysaccharides should be considered, except for cellulose and chitin. To improve the water insolubility of the materials, the chemical cross-linking or hydrophobization of polysaccharide molecules is generally conducted,^16−21^ leading to the loss of their intrinsic biochemical properties. Owing to this background, the fabrication of structural materials using polyion complexes (PICs) that consist of oppositely charged polysaccharides has attracted attention. The formation of PICs by noncovalent electrostatic interactions provides water-insoluble materials, even if water-soluble polysaccharides are used, and the remaining properties of the raw polysaccharide species. Film materials obtained by layer-by-layer (LbL) assemblies are PIC films and are widely applied.^22−28^ Further, the microcapsules and fibers of PICs have been developed.^29−34^ Conversely, we have recently succeeded in producing water-insoluble, free-standing thin films from polysaccharide PICs using hot-press techniques.^35−37^ The polysaccharide composite films swell slightly but are insoluble in pure water, whereas they swell significantly and can be partially soluble in physiological buffers at relatively high temperatures. The films possess sufficient mechanical strength for use as structural materials in a dried state. Highly flexible films are obtained in the swollen state. The mechanical strength of the films can be controlled using fillers or support films.^38,39^ The films can load and release model drugs,^40^ and their molecular permeabilities have been evaluated.^41,42^ Films of different combinations of anionic and cationic polysaccharides are readily obtained. Some physical properties are affected by polysaccharide species, whereas their macroscopic and microscopic morphologies are similar. These films are useful as cell scaffolds, and the results demonstrate that cell behavior on these films can be controlled by the polysaccharide species of the films.^43^ These findings support that these films are promising materials for biomedical applications and biodegradable structural materials for daily use.

Although various PICs have been developed and some of them are used in practical applications, the processing of PICs to obtain materials of various shapes has not been widely investigated. The topic attracts polymer chemists, and it has not been well clarified whether various shape-forming processes involve the rearrangement of the molecular chains and/or ion complex formation in PICs. Schlenoff et al. have demonstrated that the PICs of synthetic polymers can be plasticized by salt solution treatments.^44−46^ They have proposed the concept of “saloplastic” and showed that the plasticized PICs can be formed into various shapes. Such a property was also demonstrated by de Vos et al.^47^ Further, Mano et al. have reported the salt plasticization of the PICs of natural polysaccharides to obtain condensed, coacervate-type processed PICs.^48^ Additionally, Gong et al. have demonstrated that a PIC-type hydrogel can be plasticized by salt treatments, and the resulting gels can be reprocessed to various shapes with high mechanical strength.^49^ These examples are mainly focused on the PICs of hydrogel states.

Various roll-press techniques have been widely used for film fabrication from polymers. This study focuses on the availability of roll-press techniques for preparing free-standing polysaccharide composite films. For the formation of general synthetic polymers, such as engineering plastics, using thermal stretching, mechanically anisotropic films can be obtained by stretching and aligning polymer chains along the direction of stretching.^50−53^ Here, the intermolecular (interchain) interactions of polymer molecules in engineering plastics are van der Waals interactions. Contrarily, the intermolecular interactions in PICs are mainly multivalent electrostatic interactions. Therefore, the situation is completely different between the thermal stretching of engineering plastics and PICs. If multivalent electrostatic interactions are too strong, PICs hardly form homogeneous thin films under the conditions used for conventional polymers. It is worth investigating whether the general thermal stretching process can provide sufficient mechanical stress to PICs formed by multivalent electrostatic interactions to make them thin films.

In this study, we demonstrate that PICs made of natural polysaccharides can be formed into thin films using conventional roll-press techniques. The effects of the process parameters on the film formability are evaluated to determine the optimal conditions. The obtained films exhibit mechanical anisotropy along the rolling direction and anisotropic deformation properties when immersing in aqueous solutions. These properties are only derived from the roll-press process and are not observed for conventional hot-pressed films. Further, a possible mechanism for film formation is discussed.

## 2 Experimental Section

### 2.1 Materials

CHI with a low molecular weight (*M*_W_) (low *M*_W_, 50,000–190,000 based on viscosity, 20–300 cps (1% in 1% acetic acid), degree of deacetylation (DD) ≥75%, denoted as low CHI) and that of a relatively high *M*_W_ (medium *M*_W_, 200–800 cps (1% in 1% acetic acid), degree of deacetylation (DD) 75–85%, denoted as high CHI) were purchased from Sigma-Aldrich. Chondroitin sulfate C (sodium salt, from shark cartilage, *M*_W_ ca. 20,000, denoted as CS), CHI (from crab shell, *M*_W_ ≥ 100,000, degree of deacetylation (DD) 90.2%, denoted as middle CHI), and other chemicals were obtained from Nacalai Tesque Inc. The chemical structures of the polysaccharides are shown in Figure S1. All chemicals were used as received. Distilled water and ultrapure water (18.2 MΩ·cm) were prepared for the experiments (RFD210TA and RFU414BA, respectively; Advantec Toyo Kaisha, Ltd.).

### 2.2 Preparation of Roll-Pressed Films

**Figure 1:**
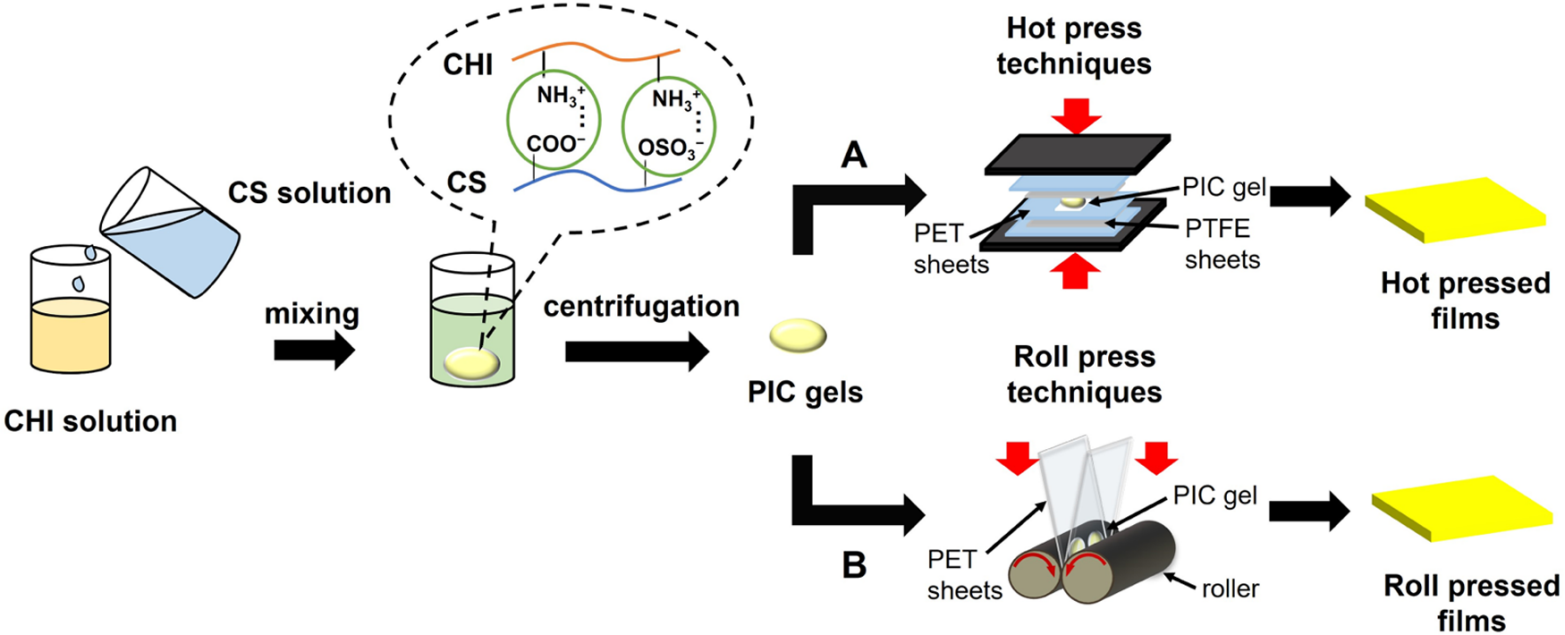
Schematic illustration of the preparation of composite films from CS/CHI PICs using hot-press techniques (A) previous studies^35,36^ and roll-press techniques (B) this study.

The film preparation process is schematically shown in Figure 1. First, polysaccharide PICs were prepared. The aqueous solutions of CS (2.0 wt % as sodium salts) were added dropwise to the aqueous acetic acid (1.0 wt %) solutions of CHI (low CHI, middle CHI, and high CHI; 1.0 wt %) until gel-like PICs were completely formed. Hereinafter, the PICs consisting of CS and CHI are denoted as CS/CHI gels. After washing with distilled water, the PICs were isolated by centrifugation (6000 rpm for 10 min).

The PIC gels obtained were used for roll pressing. A hot-rolling machine (IMC-1107, Imoto Machinery Co., Ltd.) was used. The PIC gels were sandwiched between poly(ethylene terephthalate) (PET) sheets (Lumilar, 100 μm thickness; PANAC Co.) and passed through the gap between the rollers (ϕ40 mm diameter) of the apparatus. In this study, the temperature (100–160 °C), rolling speed (2–20 rpm), inter-roller gap (450–200 μm), and repetition number were the parameters of the preparation condition. The last was determined by the appearance of the stretched gels for each condition; in other words, roll pressing was repeated until the appearance of the stretched gels did not change. The representative conditions are shown in the Supporting Information. The resulting films made from the CS/CHI gels are denoted as CS/CHI films.

Additionally, for comparison, hot-pressed films were prepared from CS/middle CHI PIC gels according to a previously reported procedure.^35,36^

### 2.3 Characterization

Fourier-transform infrared spectroscopy (FT-IR) measurements were conducted (Nicolet 380; Thermo Fisher Scientific Inc.). The spectra were obtained using the single reflection attenuation total-reflection (ATR) method with a Quest ATR accessory (GS-10800, Specac Ltd.). The X-ray diffraction (XRD) patterns of the samples were obtained using an ULTIMA IV (Rigaku Corp.) with a Cu Kα source and high-speed detector under the following conditions: tube voltage of 40 kV, tube current of 40 mA, and step width of 0.02°. The XRD patterns were obtained after subtracting the background. The morphology and elemental composition of the films were evaluated using scanning electron microscopy and energy-dispersive X-ray spectroscopy (SEM and EDX, JSM-7001F; JEOL Ltd.) with an acceleration voltage of 10 kV. In some cases, the specimens were coated with Pt–Pd using an ion-sputtering device (MC1000; Hitachi Ltd.) to prevent charge-up.

The mechanical properties of the films were quantitatively evaluated to calculate the tensile strength using a universal tester (Autograph AGS-500NJ; Shimadzu Co.). First, the film thickness required to calculate the tensile strength (MPa) was measured using a micrometer (MDE-25MJ; Mitutoyo Co.). Afterward, the films were cut into strips (1 cm × 3 cm) and placed in the apparatus while maintaining an initial gauge length of 2 cm. The stretching speed was set at 1 mm/min. The obtained stress–strain curves were analyzed using Trapezium X software (Shimadzu Co.). The maximum tensile strengths (MPa) were expressed as the mean ± S.D.

The interference colors of the films were macroscopically observed under cross-Nicol conditions, where the two polarizing sheets (Artec Co., Ltd.) were orthogonal. Further, the films were observed using an optical microscope (LV100, Nikon Co.) attached to an FL analyzer (LV-FLAN, Nikon Co.) and a simple polarizer (C-SP, Nikon Co.).

The swelling behavior of the films was evaluated according to the methods described in previous studies.^36^ The films (1 cm × 1 cm) were immersed in distilled water and incubated at room temperature. The degree of swelling (%) and weight loss (%) were calculated using eqs 1 and 2, respectively.
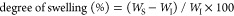
1
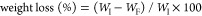
2where *W*_I_ is the initial weight of the film before immersion, *W*_S_ is the weight of the swollen film after immersion, and *W*_F_ is the final weight of the film after the immersion experiments, followed by drying in air. *W*_S_ was obtained after every 10 min of immersion.

## 3 Results and Discussion

### 3.1 Fabrication of CS/CHI Composite Films by Roll-Press Techniques

PIC gels with CS and CHI with three different *M*_W_ (low CHI, middle CHI, and high CHI) were prepared. The gels were formed by mixing the CS aqueous solution and the CHI solution. When using low CHI, the gel was softer than that of middle and high CHI. This might have resulted from the relatively low entanglement of polymer chains with low-*M*_W_ polysaccharide chains.

The obtained CS/CHI PIC gels were used to prepare the corresponding films using roll-press techniques (Figure 1). An example of the case of PIC gels consisting of CS and middle CHI (CS/middle CHI gels) is shown with the change in the macroscopic appearance of the sample, as shown in Figure S2. The centrifuged gels were sandwiched between PET sheets with a thickness of 100 μm and passed through the gap of the rollers, which were rotated at 8 rpm and heated at 120 °C, of the hot-roll press apparatus (Figure S2a). During the first stretching, the gels formed film-like structures. They were opaque and contained numerous cracks (Figure S2b). To fabricate smooth composite films, four additional cycles of roll-press treatments were carried out by decreasing the roll gap of the apparatus (Figure S2c–f and the corresponding descriptions). Five stretching cycles of CS/CHI gels yielded thin and transparent films (Figure S2f). The films were pale yellow because of the formation of saccharide byproducts (humins, difficult to characterize by spectroscopic measurements) by the Maillard reaction, which is the reaction of NH_2_ groups in CHI with the reducing ends of the polysaccharides.^54^ In a control experiment, films were prepared from lyophilized CS/CHI gels by roll pressing. The stretchability of the gels was poor, and the resulting films had many cracks (Figure S3). This result indicates that the incorporation of water into CS/CHI gels, according to the result of gel isolation by centrifugation, was essential for fabricating homogeneous films by hot-roll-press techniques.

To optimize the preparation conditions of the composite films by roll-press techniques, the effect of roll speed was first investigated. Composite films with different rolling speeds were prepared at 120 °C using CHI of three kinds of *M*_W_ (low CHI, middle CHI, and high CHI) (Figure S4). The resulting films with CHI were transparent at a rolling speed of 2 rpm. Conversely, the composite films obtained using rolling speeds of 8 and 20 rpm were opaque owing to scattering, indicating the formation of inner micropores by the rapid and inhomogeneous evaporation of water during relatively fast hot-roll pressing. At 2 rpm, almost all of the incorporated water in the PIC gels evaporated after several stretching cycles, which afforded dense composite films without cracking by the evaporation of water. Further, this tendency was observed for films with different chain lengths of CHI (high and low CHI). The yellow color of the films prepared at 2 rpm was deeper than that of the films prepared at 8 and 20 rpm. This indicates that the Maillard reaction proceeded more with long exposure to high temperatures because of the relatively low rolling speeds. The thicknesses of the films were measured (Figure S5A). The thickness slightly increased with the increase in rolling temperature. This means that a high rolling speed results in reduced gel stretching. However, the thicknesses of the films with different *M*_W_ of CHI were comparable when prepared at the same rolling speed, indicating no significant effect of the *M*_W_ of CHI on the film thickness. Further, the films were also prepared at rolling speeds of 4 and 12 rpm. The tendencies of the number of cracks and yellow color intensity of the films prepared at 4 and 12 rpm were between the results for films prepared at 2 and 8 rpm and between the results for the films prepared at 8 rpm and 20 rpm, respectively.

**Figure 2:**
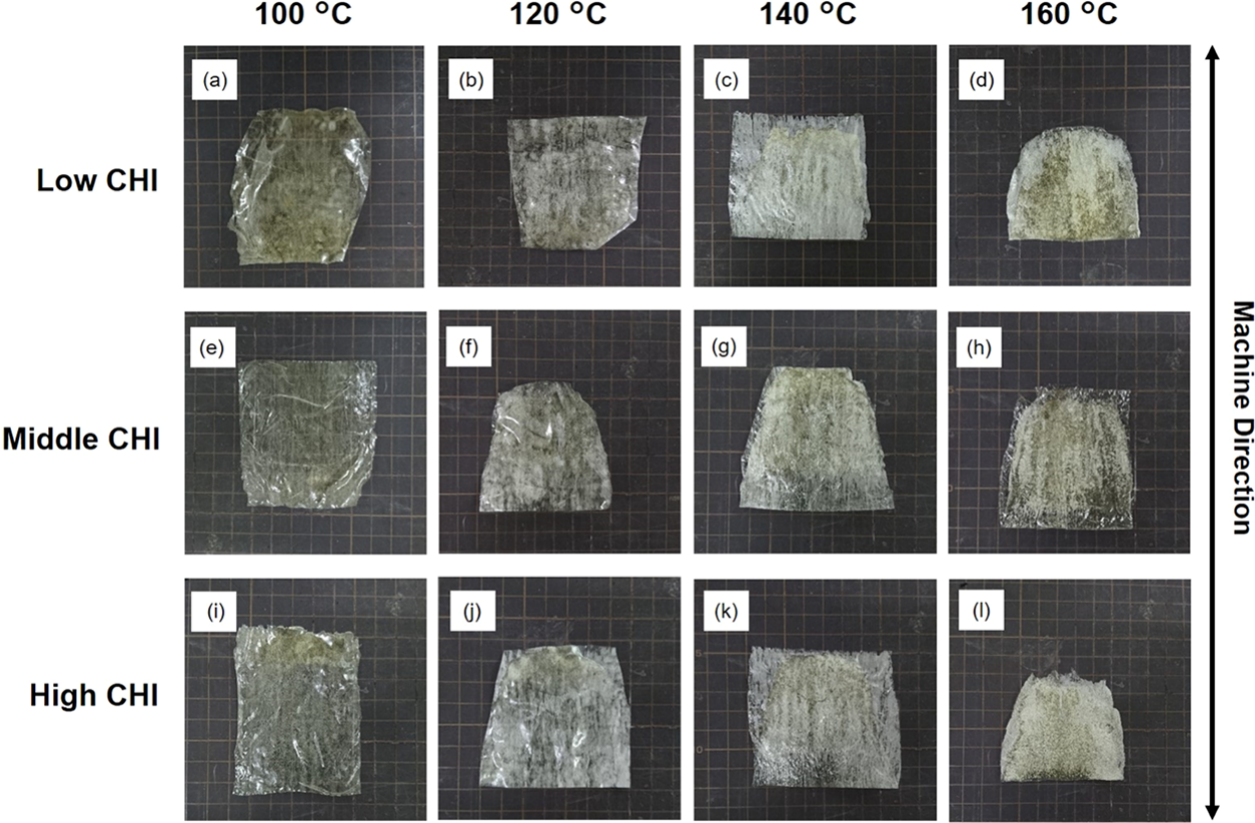
Photographs of roll-pressed films prepared using three kinds of CHI (Low CHI (a–d), middle CHI (e–h), and high CHI (i–l)) at the rolling speed of 8 rpm and different roll temperatures; the unit grid size is 1 cm × 1 cm.

Next, the effect of the roll temperature on the film formability was examined. The morphologies of the films prepared at different temperatures (100, 120, 140, and 160 °C) at 8 rpm are shown in Figure 2. The film prepared at 100 °C was smooth and transparent with each kind of *M*_W_ of CHI (Figure 2a,e,i). When the roll temperature was 120 °C or higher, the scattering of the films as a result of the inner micropores increased owing to the rapid evaporation of the incorporated water in the films during roll pressing (Figure 2b–d,f–h,j–l). In particular, at roll temperatures of 140 and 160 °C, the films were harder than those obtained at relatively low temperatures. These films were colored deeper yellow than those at low temperatures, indicating more progression of the Maillard reaction. The thicknesses of these films (Figure S5B) showed that there was no significant effect on the MW of CHI. Contrarily, high roll temperatures increased the film thickness. These results suggest that the fast water evaporation made the polysaccharide PIC gels less stretchable.

### 3.2 Characterization of the Roll-Pressed Films

**Figure 3:**
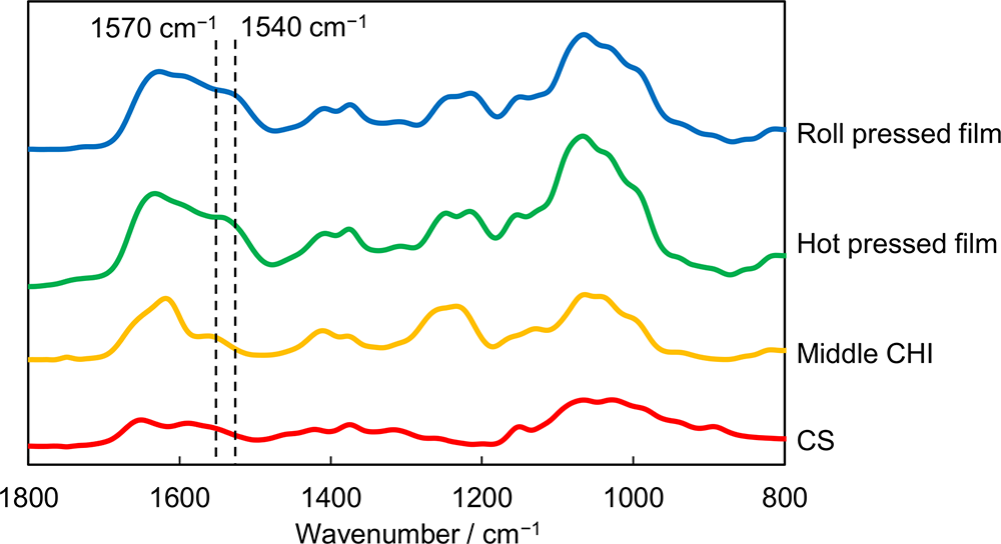
FT-IR spectra of the CS/CHI films and each polysaccharide.

**Figure 4:**
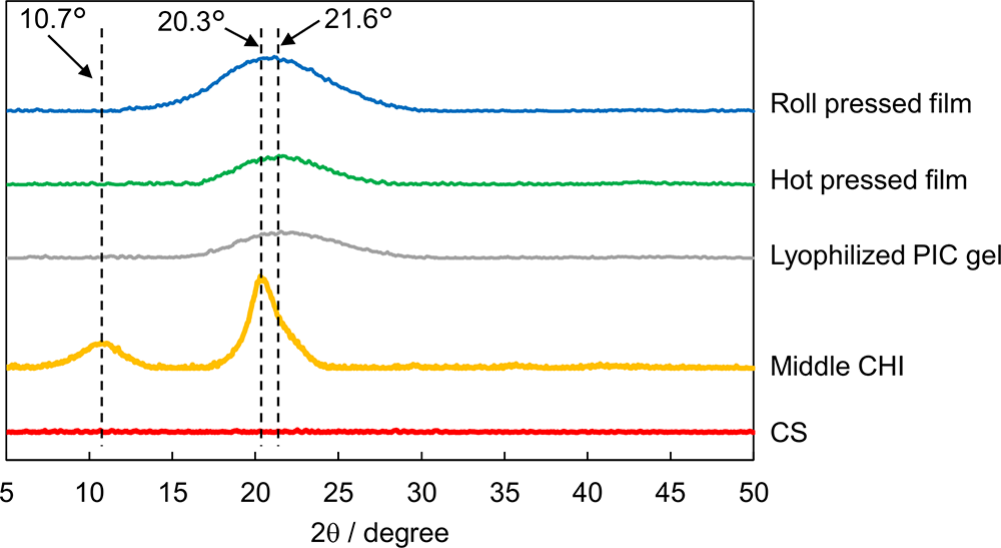
XRD patterns of the CS/CHI films, PIC gel, and each polysaccharide.

To clarify the differences in the structural properties of the composite films prepared using the hot-press technique^35,36^ and the roll-press technique, the FT-IR spectra and XRD patterns of these films (CS/middle CHI films) were obtained. The FT-IR spectra of the roll-pressed films and the hot-pressed films were comparable, and the peaks at 1540 cm^–1^ in these spectra were assignable to the N–H bending of the protonated amine groups in CHI interacting with the anionic charged groups (COO^–^ and SO_3_^–^) in CS (Figure 3).^33^ The middle CHI has unprotonated amino groups and appears around 1570 cm^–1^. When the amino group is protonated, the NH bending vibration is red-shifted and the wavenumber is to be smaller. It indicated that the preparation techniques of the composite films did not affect the chemical structure and electrostatic interactions between CS and CHI in the films. The XRD patterns of the films, CS, CHI, and CS/CHI gels were measured to characterize the crystalline phases of the films and polysaccharides (Figure 4). In the diffraction pattern of CHI, the peaks at 10.7 and 20.3° were assignable to (002) and (200) diffractions, respectively, according to a previous report.^55^ Conversely, the diffraction pattern of CS had no peaks, indicating that CS was amorphous. A new broad peak appeared at 21.6° in the diffraction pattern of the PIC gels obtained by mixing CS and CHI solutions. Although the origin of this peak has not been identified, the disappearance of the diffraction peaks of CHI and the appearance of the new peak indicated the disassembly of the crystalline CHI structures and the formation of a new structure with a specific crystallinity originating from CS/CHI PICs. In the XRD patterns of the hot-pressed and roll-pressed films prepared at 120 °C, broad peaks at 21.6° appeared, similar to that of the PIC gels. These results indicate that the pressing method had no significant effect on the internally ordered structures of the resulting films at the molecular level, and the structures were essentially the same as those of the PICs before pressing.

**Figure 5:**
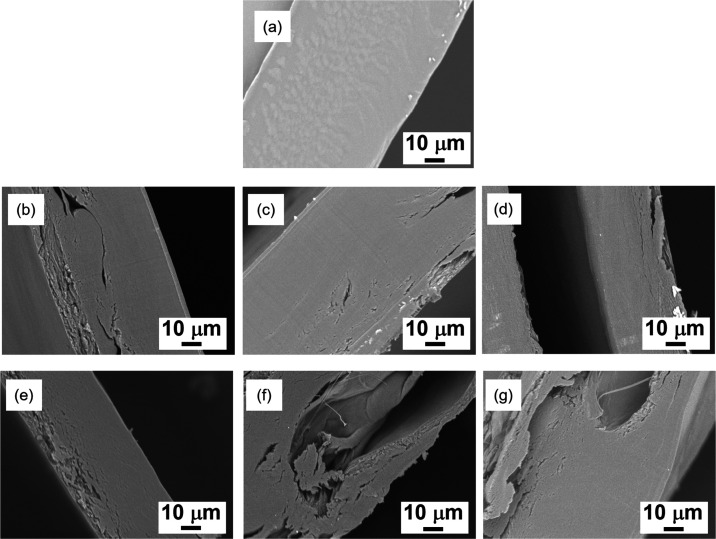
Cross-section SEM images of the CS/middle CHI films; (a) hot-pressed films (120 °C); (b–d) roll-pressed films prepared at 120 °C and the rolling speed of 2 rpm (b), 8 rpm (c), and 20 rpm (d); and (e–f) roll-pressed films prepared at the rolling speed of 8 rpm and 100 °C (e), 140 °C (f), and 160 °C (g).

The SEM observations of the roll-pressed films prepared using different roll speeds and temperatures were performed to evaluate their microscopic morphologies (Figure 5). The SEM images of a cross-section of the hot-pressed films showed smooth and dense structures without cracks and pores (Figure 5a). Contrarily, the images of the roll-pressed films revealed that all films had many cracks and heterogeneous layer structures. These results were reasonable because the films were prepared by roll pressing the folded films (see the description for Figure S2). Ideally, the layered structures should disappear after effective roll pressing. The present results indicate that the pressure supplied by the two rollers of the apparatus was not sufficient to produce microscopically homogeneous films. This is a limitation of using tabletop laboratory-scale apparatus. The quality of the films can be improved on an industrial scale, which makes it possible to apply high pressure to the rolling films. Additionally, the SEM results revealed that high roll speed and temperature resulted in more cracks and pores than those at low roll speed and temperature (Figure 5d,f,g), indicating the reduced stretchability of the PIC gels under these conditions. This trend corresponds to the results of the macroscopic film formability and mechanical properties.

### 3.3 Anisotropic Properties of the Roll-Pressed Films

**Figure 6:**
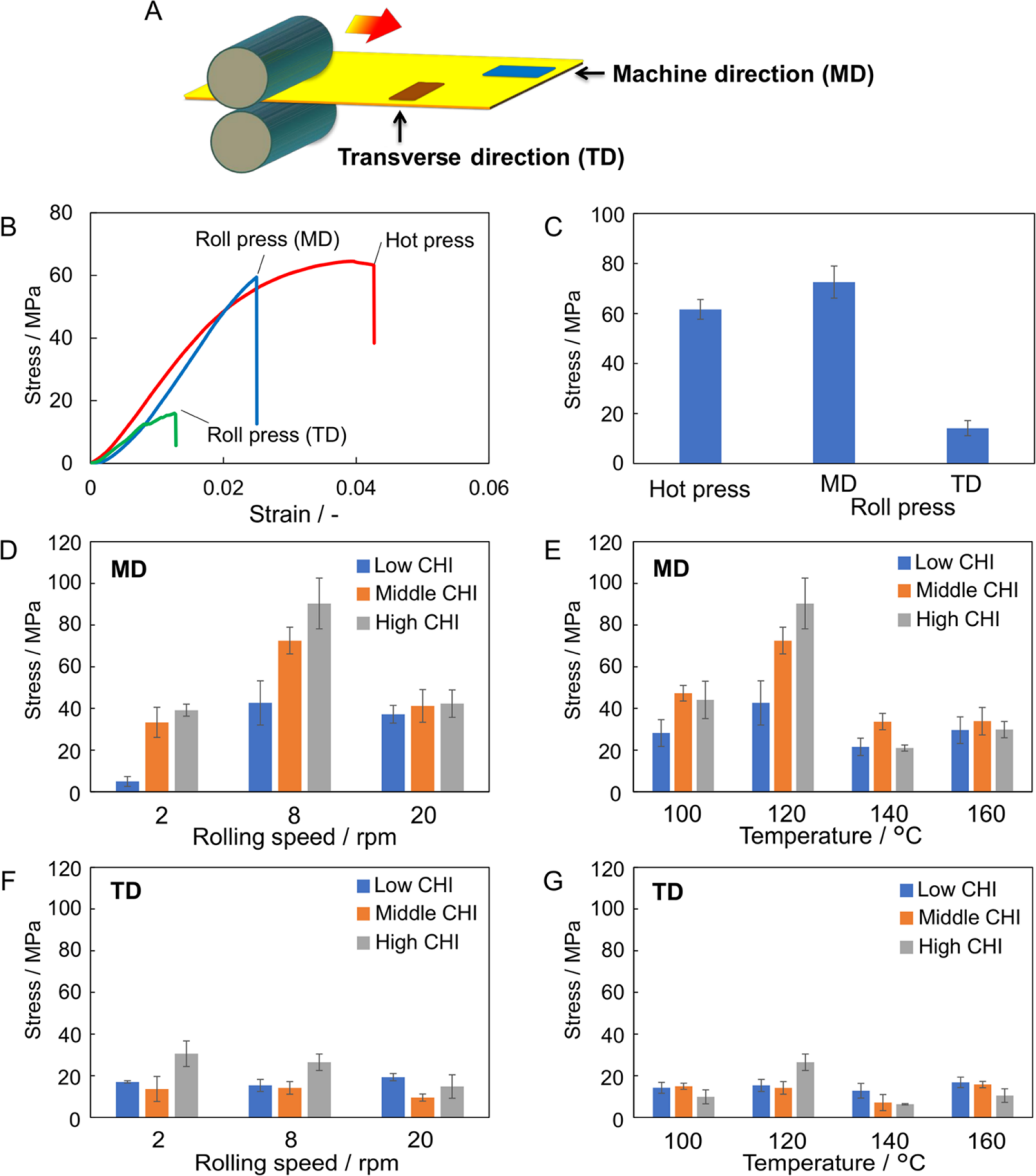
Tensile strength of the CS/CHI composite films; (A) schematic illustration of the relationship between the cutting direction of the sample strips and the rolling direction of the films; (B) typical stress–strain curves of hot-pressed films, the MD of roll-pressed films, and the TD of roll-pressed films (CS/middle CHI films, hot press: 120 °C, roll press: 2 rpm, 120 °C); (C) comparison of the maximum stress among the hot-pressed films, the MD of roll-pressed films, and the TD of roll-pressed films (CS/middle CHI films, hot press: 120 °C, roll press: 8 rpm, 120 °C); and (D–G) maximum stress of the MD (D, E) and TD (F, G) of the films prepared at 120 °C and different rolling speeds (D, F) and that at the rolling speed of 8 rpm and different temperatures (E, G).

To clarify the mechanical properties of the roll-pressed films, the tensile strength of these films was measured using different fabrication techniques (Figure 6). The directions parallel and perpendicular to the rolling direction are denoted as the mechanical direction (MD) and transverse direction (TD), respectively (Figure 6A). Typical stress–strain curves of hot-pressed films, the MD of the roll-pressed film, and the TD of roll-pressed films are shown in Figure 6B. For the CS/middle CHI films, the tensile strength of the MD of the roll-pressed films (72.6 ± 6.4 MPa) was slightly higher than that of the hot-pressed film (61.6 ± 3.9 MPa, Figure 6C). Conversely, the tensile strength of the TD of the roll-pressed films (14.2 ± 3.0 MPa) was considerably smaller than that of the MD samples, which showed that the roll-pressed films had mechanical anisotropy. The tensile strength of the roll-pressed films prepared with different roll speeds, roll temperatures, and MW of CHI were determined to elucidate the effects of these parameters on the mechanical properties of the films (Figure 6D–G). In general, the tensile strength of the MD of the roll-pressed films appeared larger than that of the TD of the film under the present conditions. Regarding the effect of the rolling speed, 8 rpm was the best for obtaining good films among the three conditions (Figure 6D,F). At a rolling speed of 8 rpm, the MD of the CS/high CHI films showed the highest tensile strength of 90.3 ± 12.1 MPa. In contrast, the CS/middle CHI films exhibited the highest mechanical anisotropy (MD/TD = 4.9). The rolling speed of 2 rpm seemed to be insufficient to elongate the inner polysaccharide chains, which might reflect the low tensile strength of the MD of the CS/low CHI films. At a rolling speed of 20 rpm, many pores and cracks were observed in the films by SEM (Figure 5d). Such microstructures in the films were one possible reason for the lower tensile strength of the MD of the films than that obtained at 8 rpm and the lack of dependence of the *M*_W_ of CHI on the tensile strength. For the effect of roll temperature, the highest tensile strength and mechanical anisotropy were obtained for CS/high CHI and CS/middle CHI films prepared at 120 °C, respectively (Figure 6E,G). The mechanical properties of the films improved upon increasing the roll temperature from 100 to 120 °C, whereas the films prepared at 140 or 160 °C showed relatively low mechanical strength because of the many cracks and pores in the films resulting from the rapid evaporation of incorporated water at relatively high temperatures (Figure 5f,g). Accordingly, the roll-pressed CS/middle CHI films prepared at 8 rpm and 120 °C exhibited good tensile strength and the highest mechanical anisotropy.

**Figure 7:**
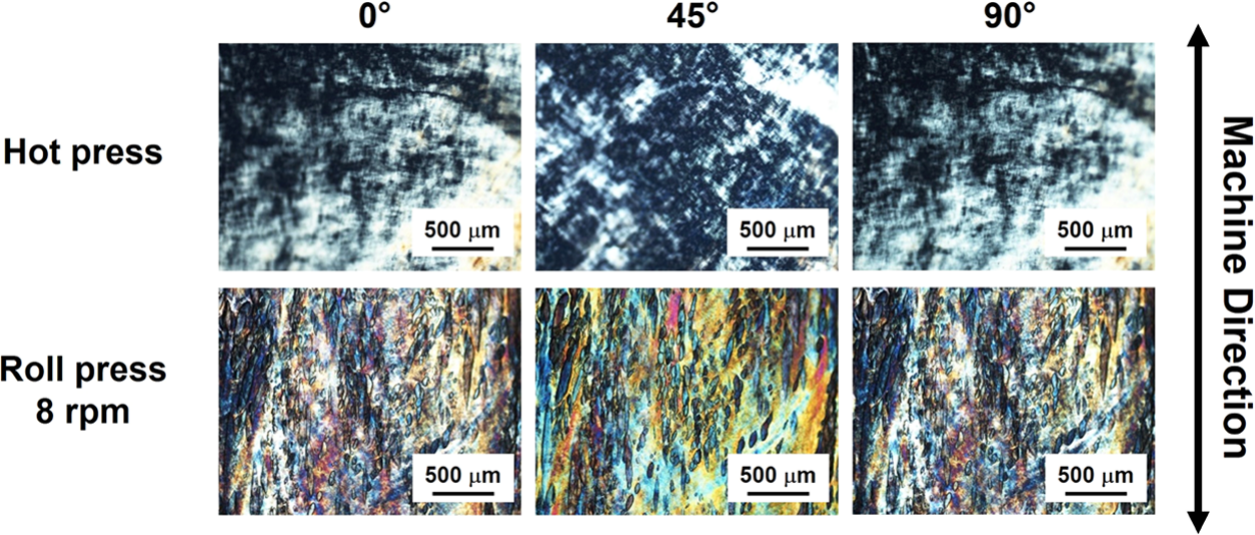
Polarized optically microscopic observation of the hot-pressed films and the roll-pressed films.

**Figure 8:**
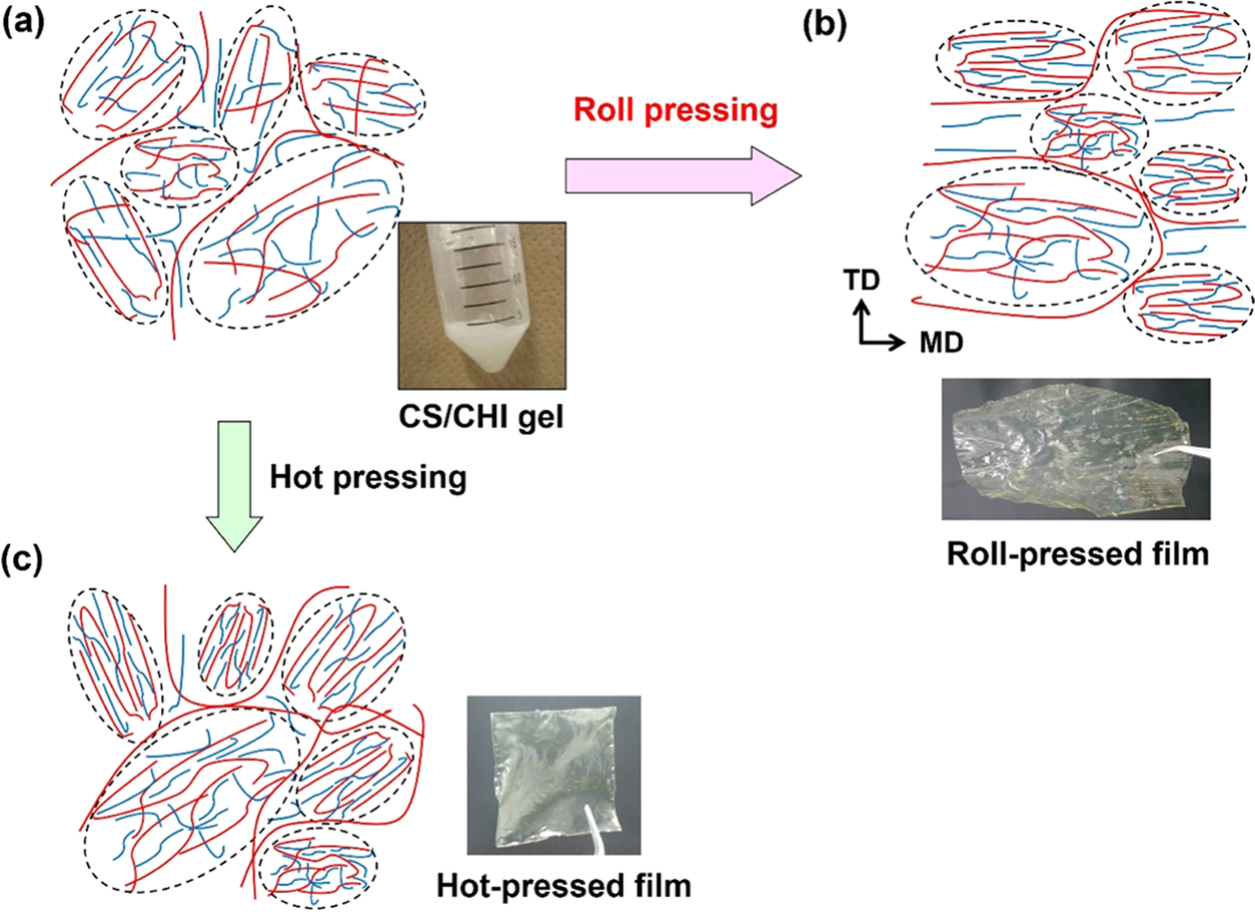
Illustration of a presumable mechanism of how the roll-pressed films obtain structural anisotropies; (a) CS/CHI gel; (b) roll-pressed film; and (c) hot-pressed film.

To evaluate whether the mechanical anisotropy of the roll-pressed films was due to their structural anisotropy, the interference colors of the films were observed. The films prepared at a rolling speed of 8 rpm and 120 °C that exhibited the highest mechanical anisotropy were examined, and the hot-pressed films were used as a control. In the macroscopic observations under cross-Nicol conditions, the roll-pressed films showed interference color, indicating that the films were anisotropic (Figure S6). However, the hot-pressed films exhibited no interference color during the same observation. These results support the idea that roll-press techniques induced the molecular orientation of polysaccharides along the MD in the films. Further, the interference colors of the roll-pressed films were confirmed by polarized microscopic observations (Figure 7). The colors were changed by rotating the films from 45 to 90°. The hot-pressed film exhibited no interference color under the same conditions. It was difficult to conduct quantitative evaluation, such as obtaining the parameters of optical retardation, because the in-plane color distributions in the films were complicated.^56^ However, the results of these observations suggested that the mechanical anisotropies of the roll-pressed films were caused by their structural anisotropies. According to the XRD results, the degree of ordered assembly of the molecules in the films was similar for the hot-pressed and roll-pressed films (Figure 4). Therefore, the results of the interference colors of the films might reflect the difference in the ordering of unit (domain) structures with specific crystalline-like structures (see the discussion below and Figure 8).

**Figure 9:**
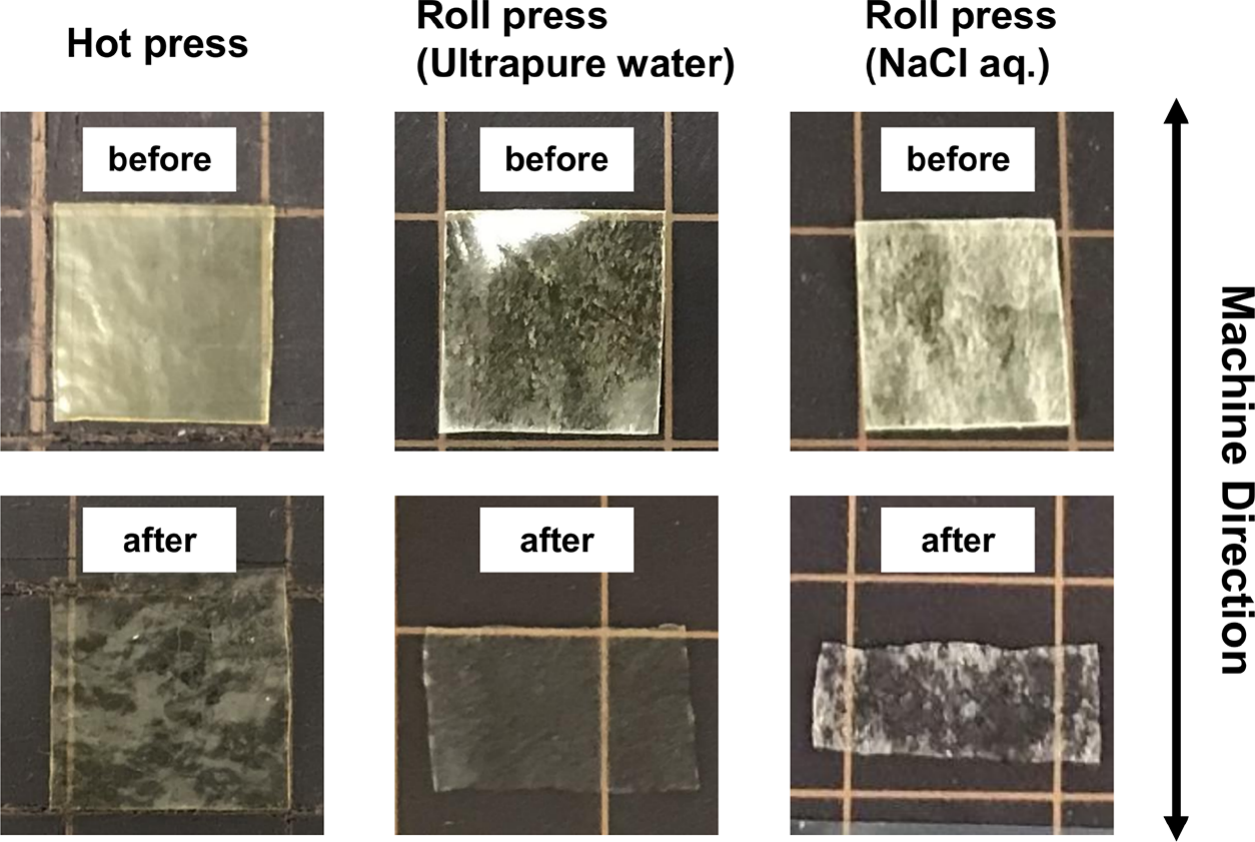
Swelling behavior of hot-pressed films in ultrapure water and roll-pressed films in ultrapure water or NaCl aqueous solutions; the unit grid size is 1 cm × 1 cm.

Additionally, the swelling behaviors of the roll-pressed films in ultrapure water and NaCl aqueous solutions were compared with those of the hot-pressed films (Figure 9). For the hot-pressed films, the films expanded in both directions parallel and perpendicular to the arrows shown in Figure 9 with a similar ratio (parallel: 13 ± 3.9%, perpendicular: 13 ± 4.2%) after immersion in ultrapure water for 20 min (Figure 9, left). Conversely, the roll-pressed films showed expansion in the TD (perpendicular to the arrow in Figure 9) (15 ± 3.9%) after immersion in ultrapure water for 20 min (Figure 9, middle). Notably, the films shrank by 10 ± 5.5% in MD upon immersion in ultrapure water. Such anisotropic responses to water immersion support the structural anisotropies of the roll-pressed films. The PIC gels were stretched to MD by roll pressing, which accompanied the solidification of the resulting films by the evaporation of water. After the samples were cooled to room temperature, the stretched and tensioned structures were fixed in the films via multivalent hydrogen bonding and electrostatic interactions among the polysaccharides. When immersing the films in water, the tensioned structures were relaxed by incorporating water inside the films, which partially broke the hydrogen bonds, resulting in a large shrinkage of the films in the MD. The immersion of the roll-pressed films in 0.2 M NaCl solution for 20 min afforded higher shrinking in MD (24.0 ± 7.7%) and expansion in TD (29.0 ± 4.5%) than that in ultrapure water (Figure 9, right). NaCl solutions can inhibit hydrogen bonding electrostatic interactions among polysaccharides. The latter point should contribute to the higher swelling of the films compared to that in ultrapure water. Additionally, it has been reported that salt treatments rearranged the PIC structures in the gels.^44^ These factors resulted in the relatively high shrinking in MD and expansion in TD for the films. Accordingly, the immersion of the roll-pressed films into an aqueous solution reoriented the polysaccharide chains in the films. These results support the stretching effect of the present roll-press techniques.

As mentioned above, the XRD and FT-IR studies indicated that the ordering of the molecular assembly and electrostatic interactions among polysaccharides in the roll-pressed films were similar to those in the hot-pressed films (Figures 3 and 4). Nevertheless, the roll-pressed films exhibited mechanical anisotropies caused by the structural anisotropies of the films (Figures 6 and 7). A plausible mechanism for obtaining structural anisotropy by roll pressing is shown in Figure 8. The PIC gels prepared in this study may consist of two types of unit microgel structures: ordered and less-ordered structures (Figure 8a). The microstructure of the bulk PICs was not homogeneous at the molecular level because the gels were obtained by the dropwise addition of CS solutions to CHI solutions (see the Experimental Section). By roll pressing, the PIC gels were mechanically stretched; however, the applied forces were probably insufficient to induce the molecular alignment of polysaccharides in the PICs (both ordered and less-ordered structures), as supported by the XRD results (Figure 4). Resultantly, roll pressing induced the ordering of the ordered unit structures (Figure 8b). For hot pressing, the original PICs were pressed by maintaining their inner microstructures or slightly stretched from the center to the peripheral; however, the unit microgel structures were not ordered in one direction (Figure 8c). Therefore, differences in the structural anisotropy between the roll-pressed and hot-pressed films were observed.

## 4 Conclusions

This study demonstrates that roll-press techniques can produce free-standing thin films of polysaccharide PICs with structural and mechanical anisotropies. This indicates that abundant biomass, such as natural polysaccharides, can be used as more advanced functional structural materials. The films prepared using middle CHI at a rolling speed of 8 rpm and roll temperature of 120 °C exhibited the highest mechanical anisotropy. It was indicated that roll pressing induced the ordering of unit microgel structures, which afforded mechanical anisotropies. The characteristics of the roll-pressed films support their promising application in biomedical fields, such as their use as wound dressings for tendon areas or plasters for joint regions. Further, the roll-pressed films showed shrinkage in MD and expansion in TD after immersion in aqueous solutions, followed by drying. Such anisotropic shrinking and extension properties caused by water treatment indicate that these films are applicable as shape-memory materials.

## Supporting Information Available

Chemical structures of chondroitin sulfate C sodium salts (CS) and chitosan (CHI); photographs of preparation of CS/CHI films by roll-press techniques; photographs of the lyophilized gel and composite films from the gel by roll-press techniques; photographs of roll-pressed films with different rolling speeds and *M*_W_ of CHI; film thickness of roll-pressed films composed of each CHI; and macroscopic observation of interference colors of the films using two polarizing films under the cross-Nicol conditions (PDF)
