# Supplementary material for: Preparation of Mechanically Anisotropic Polysaccharide Composite Films Using Roll-Press Techniques
Source: ACS Omega. 2023 Feb 1;8(6):5607–16. doi: 10.1021/acsomega.2c07077 (PMC9933227; doi:10.1021/acsomega.2c07077)
Supplement: Supplementary file 1 — ao2c07077_si_001.pdf [file ao2c07077_si_001.pdf]

Preparation of Mechanically Anisotropic Polysaccharide Composite Films Using Roll Press Techniques

Takuya Sagawa,[†,§] *Yuichi Nikaido,*[‡] *Kazutoshi Iijima,*[†,¶] *Masahiro Sakaguchi,* [§]*Yusuke Yataka,*[†] *and Mineo Hashizume*[*,†,§,‡]

† Department of Industrial Chemistry, Faculty of Engineering, Tokyo University of Science, 6-3-1 Niijuku, Katsushika-ku, Tokyo, 125-8585, Japan

‡ Graduate School of Chemical Sciences and Technology, Tokyo University of Science,12-1 Ichigayafunagawara-machi, Shinjuku-ku, Tokyo 162-0826, Japan

§ Graduate School of Engineering, Tokyo University of Science, 6-3-1 Niijuku, Katsushika-ku, Tokyo, 125-8585, Japan

¶ Present address: Faculty of Engineering, Yokohama National University, 79-5, Tokiwadai, Hodogaya-ku, Yokohama 240-8501, Japan

KEYWORDS: Free-standing film, Polysaccharide, Polyion complex, Roll press, Mechanical anisotropy

–Totals– 6 pages, 6 figures

Table of Contents

Figure S1 Chemical structures of chondroitin sulfate C sodium salts (CS) and chitosan (CHI). ··· S2

Figure S2 Photographs of preparation of CS/CHI films by roll press techniques. ··· S3

Figure S3 Photographs of lyophilized gel and composite films form the gel by roll press techniques. ··· S4

Figure S4 Photographs of roll-pressed films with different rolling speed and MW of CHI. ··· S4

Figure S5 Film thickness of roll-pressed films composed of each CHI. ··· S5

Figure S6 Macroscopic observation of interference colors of the films using two polarizing films under the cross Nicol conditions. ··· S5

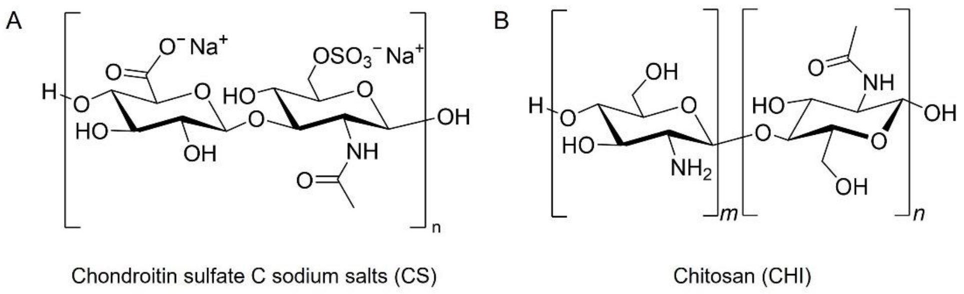

Figure S1. Chemical structures of (A) chondroitin sulfate C sodium salts (CS) and (B) chitosan (CHI).

Typical preparation method of roll pressed films from PIC gel.

As a typical experiment, fabrication of roll pressed CS/CHI films at 120 °C with rolling speed at 8 rpm from water-containing PIC gels was described. Rollers in apparatus were preheated at 120 °C, and the inter-roll gap was set to 450 μm. Then, PIC gel was sandwiched between 100 μm thickness of PET sheets and passed through the gap of rollers at 4 times (First stretching, Figure S2b). The expanded composite film was folded in a half. The gap of roller was changed to 400 μm, and the film was passed through the gap of rollers at 3 times (Second stretching, Figure S2c). The extended composite film was folded in a half. The gap was changed to 300 μm, and the film was passed through the gap of rollers at 3 times (Third stretching, Figure S2d). The obtained composite film was folded in a half. The gap was changed to 200 μm, and the film was passed through the gap at 2 times (Forth stretching, Figure S2e). The obtained composite film was folded in a half. The gap was changed to 200 μm, and the film was passed through the gap at 2 times (Forth stretching, Figure S2e). The composite film was folded in a half and passed through the gap at 2 times (Fifth stretching, Figure S2f). Finally, the PET sheets were peeled out, and the roll pressed film was obtained. Because the thickness of the PET sheet was 100 μm, the inter-roll gap of 200 μm corresponded to the situation that the thickness of the film was 0 μm. However, the objects (two PET sheets and film) with a thickness of 200 μm or more could pass through the inter-roll gap because the roller shafts were fixed by springs.

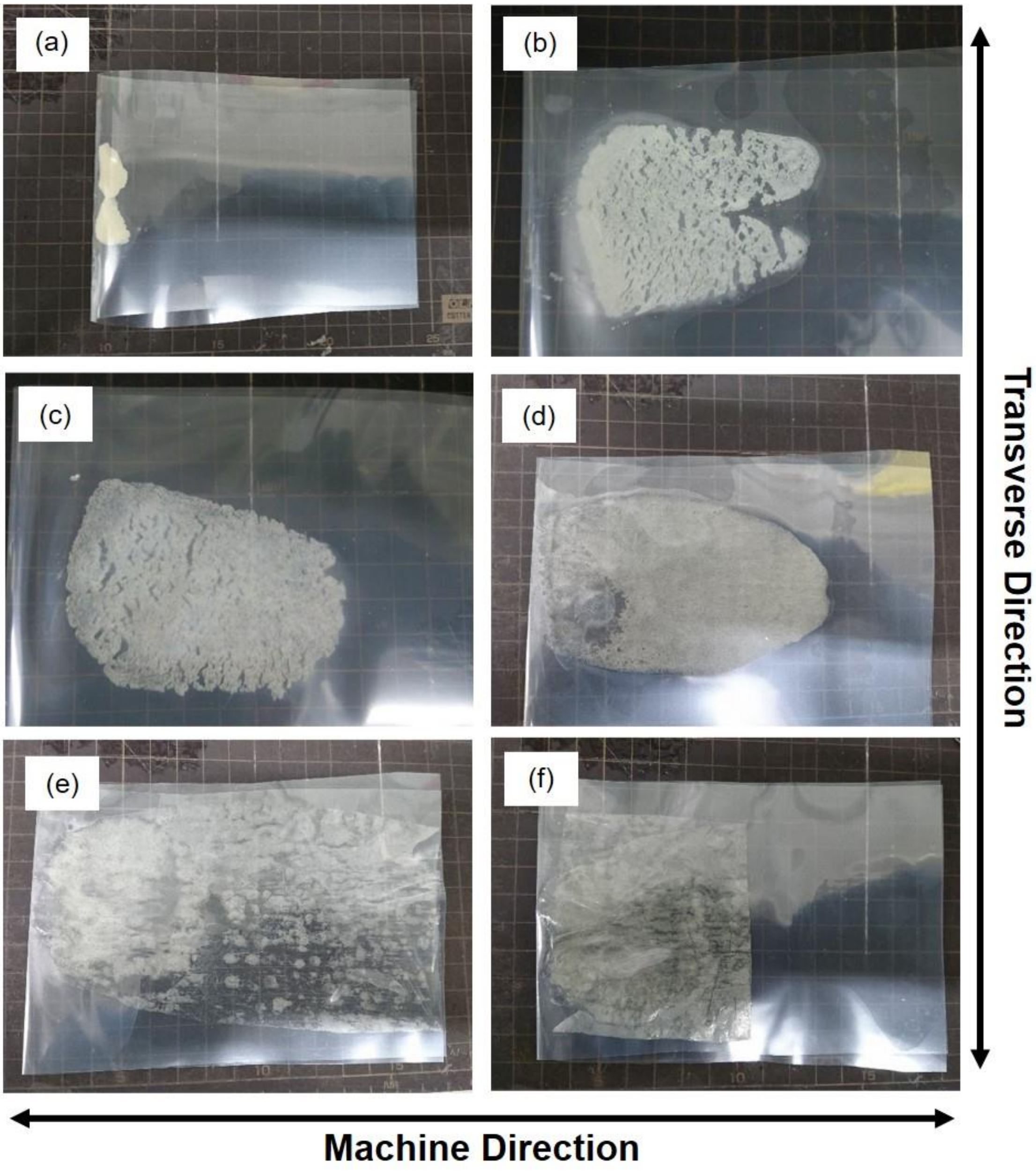

Figure S2. Photographs of preparation of CS/CHI films by roll press techniques (8 rpm, 120 °C). (a) CS/Middle CHI gel without lyophilization sandwiched between PET sheets. (b) First roll-pressed composite film. (c) Second roll-pressed composite film. (d) Third roll-pressed composite film. (e) Forth roll-pressed composite film. (f) Fifth roll-pressed composite film.

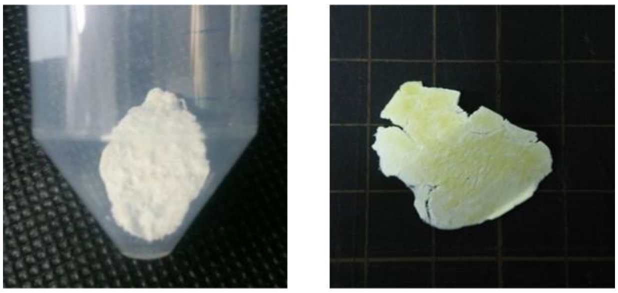

Figure S3. Photographs of lyophilized gel (left) and composite films form the gel by roll press techniques (right).

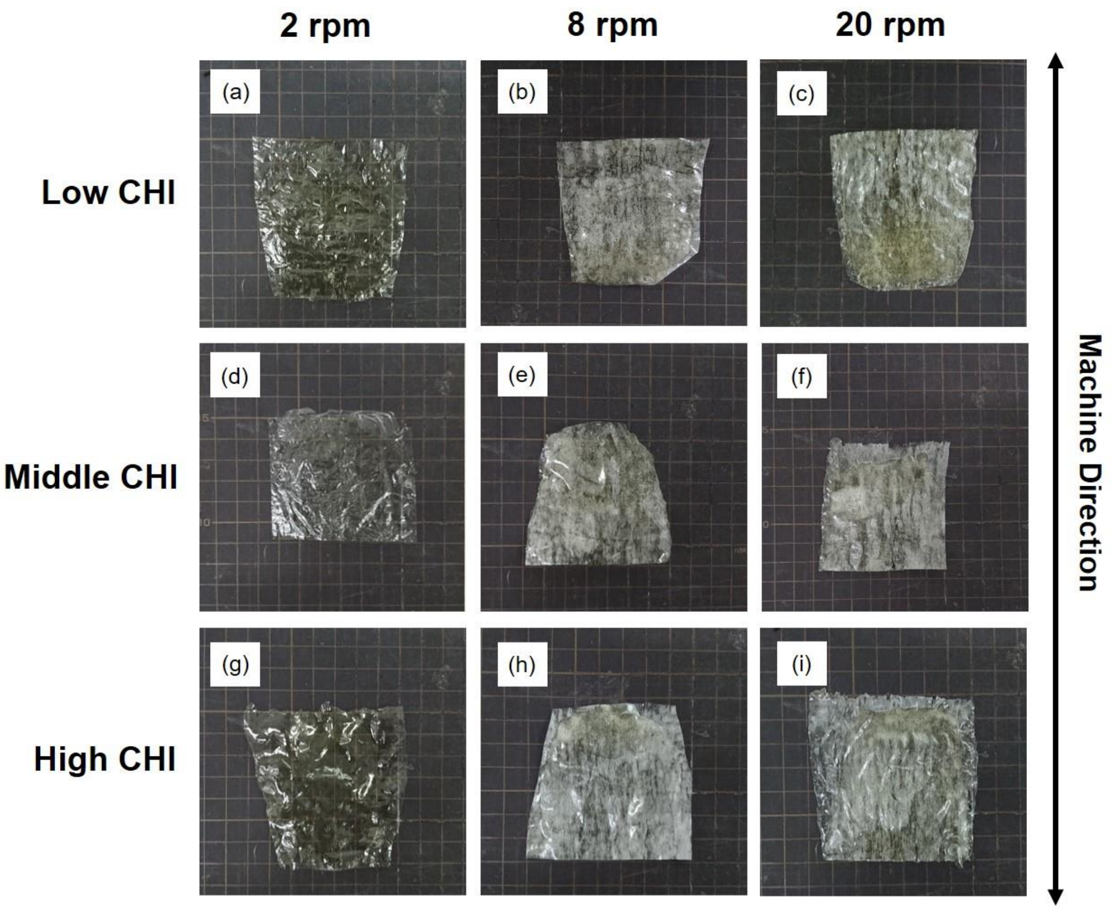

Figure S4. Photographs of roll pressed films prepared using three kinds of CHI (Low CHI (a-c); Middle CHI (d-f); High CHI (g-i)) at 120 °C and different rolling speeds.

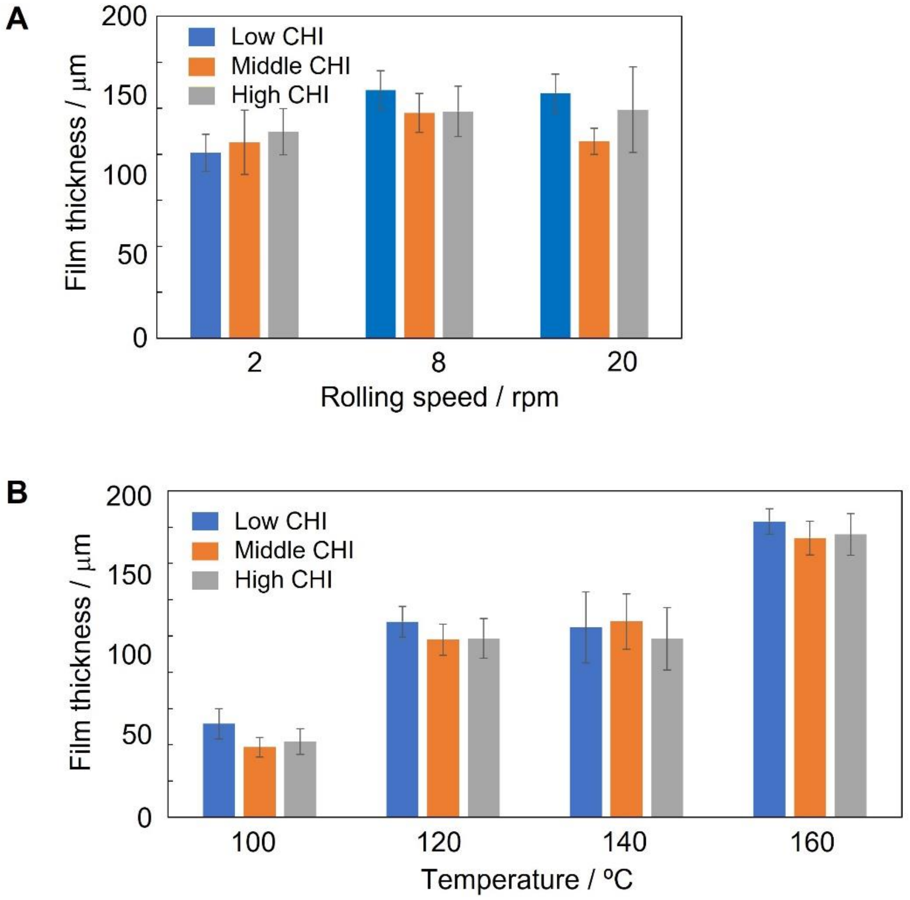

Figure S5. Film thickness of roll-pressed films prepared using different MW of CHI. (A) Prepared at 120 ºC with different rolling speed, (B) prepared at 8 rpm with different temperature.

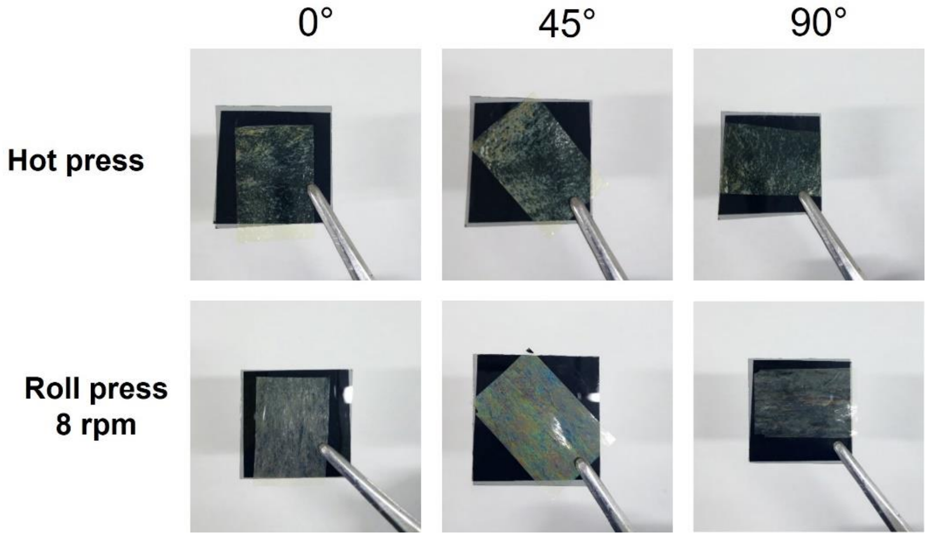

Figure S6. Macroscopic observation of interference colors of the films using two polarizing films under the cross Nicol conditions.
